# Skull-Base Inflammatory Pseudotumor Involving the Trigeminal and Facial Nerves: A Singular Presentation of a Rare Disease

**DOI:** 10.1055/s-0040-1702921

**Published:** 2020-03-03

**Authors:** Giacomo Fiacchini, Christina Cambi, Antonio Gaetano Tavoni, Luca Bruschini, Roberto Castellana, Iacopo Dallan, Stefano Berrettini

**Affiliations:** 1 Otolaryngology, Audiology, and Phoniatric Operative Unit, Department of Surgical, Medical, Molecular Pathology, and Critical Care Medicine, Azienda Ospedaliero Universitaria Pisana, University of Pisa, Pisa, Italy; 2 Clinical Immunology Unit, Department of Clinical and Experimental Medicine, Azienda Ospedaliero Universitaria Pisana, University of Pisa, Pisa, Italy; 3 Radiology Department, Azienda Ospedaliero Universitaria Pisana, University of Pisa, Pisa, Italy

**Keywords:** facial nerve, inflammatory pseudotumor, skull base, trigeminal nerve

## Abstract

Inflammatory pseudotumor (IPT) is a rare disease that is often misinterpreted as a lymphoma or carcinoma. It may involve different body regions but most commonly the lungs and the orbital cavity. We report the case of a patient affected by an IPT of the trigeminal and facial nerves.

A 69-year-old male presented to our hospital with a right facial palsy arisen suddenly 2 days before. A magnetic resonance imaging (MRI) of the head showed an abnormal mass with homogeneous enhancement involving the deep lobe of the parotid gland, the parapharyngeal space, and the infratemporal fossa, extending along the trigeminal nerve and the facial nerve. The patient was planned for multiple transnasal biopsies in the nasopharynx, the region of the foramen ovale, and the deep lobe of the parotid gland, but the results were inconclusive, with no evidence of a malignant process. We considered the possibility that the lesion could be an IPT, and the patient was treated with prednisone and cyclophosphamide. Three months after the conclusion of the treatment, an MRI showed a complete radiological response.

Inflammatory pseudotumor (IPT) is a rare disease with idiopathic pathogenesis that is often misinterpreted as a lymphoma or carcinoma due to its local aggressiveness on the surrounding structures. It may involve different body regions but most commonly it is found in the lungs and inside the orbital cavity. 1

We report the case of a patient affected by an IPT of the trigeminal and facial nerves and discuss the diagnostic and management considerations. To the best of our knowledge, this is the first report of an IPT involving both the fifth and sixth cranial nerves.

## Case Report

**Fig. 1:**
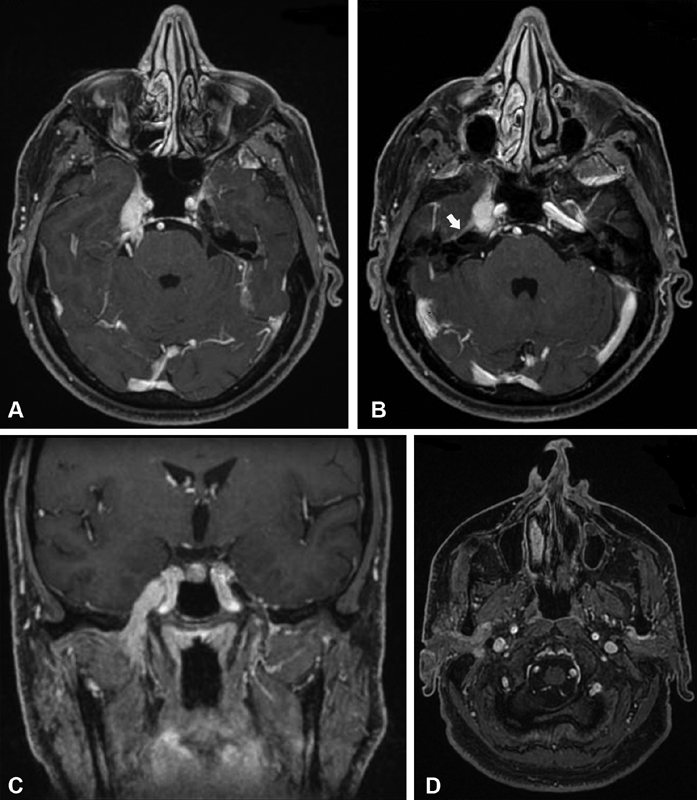
( **A,B** ) Axial and ( **C** ) coronal T1-weighted image with contrast media that demonstrate an enhanced mass lesion of the right Meckel cave, which extends posteriorly to the intracisternal portion of the fifth cranial nerve and involves the right mandibular branch of the fifth cranial nerve. Enhancement of the greater petrosal nerve and the geniculate ganglion is also present ( *white arrow* ). ( **D** ) Axial T1-weighted image with contrast media that demonstrates the involvement of the deep lobe of the right parotid gland and the upper portion of the right parapharyngeal space as well.

An otherwise healthy 69-year-old white male followed at our outpatient clinic for a bilateral sensorineural hearing loss presented to the Emergency Department of our hospital with a right facial palsy arisen suddenly 2 days before. Four months earlier, he had been diagnosed with a right trigeminal neuralgia, which was treated with gabapentin and oxcarbazepine. The initial diagnosis made at his arrival at the Emergency Department was right Bell's palsy for which he was given prednisone 50 mg for 5 days and 25 mg for the following 5 days in association with valaciclovir 500 mg twice per day for 5 days. In the following days, he showed no improvement of both the facial palsy and the trigeminal neuralgia. Laboratory work-up results were all within normal limits. At the ENT (ear, nose, and throat) evaluation, the patient showed a grade V right facial palsy according to the House–Brackmann grading system. Endoscopic evaluation of the nasal fossae, nasopharynx, and larynx showed no lesions, otoscopy revealed a right effusive otitis media, and audiological evaluation showed a right type B tympanometry, a left profound sensorineural hearing loss (diagnosed a few years before), and a right mixed hearing loss. A gadolinium-enhanced magnetic resonance imaging (MRI) of the head showed an abnormal mass with homogeneous enhancement involving the deep lobe of the right parotid gland, the upper portion of the right parapharyngeal space, the Eustachian tube, and the infratemporal fossa with both the pterygoid muscles, extending along the third division of the trigeminal nerve, through the foramen ovale, to the Meckel's cave and the intracisternal portion of the fifth cranial nerve ( Fig. 1 ). Moreover, computed tomography (CT) scan showed enlargement of the right foramen ovale and the greater petrosal nerve canal. We ordered also a positron emission tomography (PET)-CT scan that confirmed a hypermetabolic focus corresponding to the previously identified mass. The primary diagnostic hypothesis at this time was lymphoma or carcinoma of the nasopharynx or of the deep lobe of the parotid gland.

**Fig. 2:**
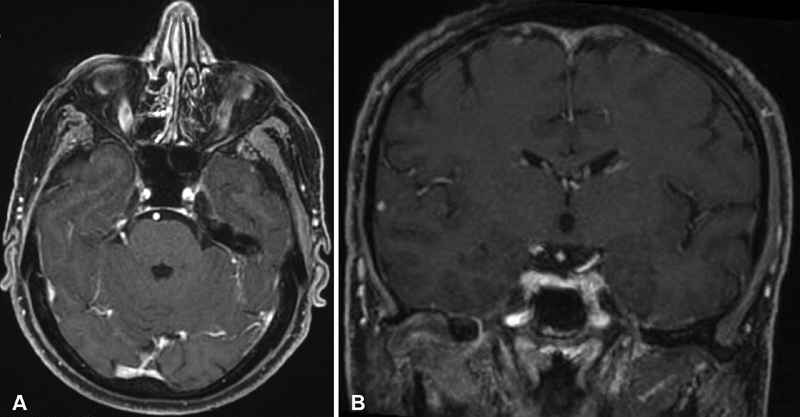
( **A** ) Axial and ( **B** ) coronal T1-weighted image with contrast media performed 3 months after therapy conclusion, demonstrating the complete resolution of the disease.

To make an accurate diagnosis, we decided to perform multiple endoscopic transnasal biopsies in the nasopharynx and the region of the right foramen ovale, just below the skull base, passing through the posterior wall of the maxillary sinus and the pterygopalatine fossa. 2 The patient was counseled about the surgical procedure and the possible side effects, and after he had signed the informed consent, the surgery was planned. The histopathological evaluation showed chronic inflammatory infiltrate with lymphoid elements T (CD3 + ) and B (CD20 + ). The proliferative activity was <10%. Few nerve fibers were also noted. Stains were negative for bacteria, fungi, and acid-fast bacilli. Tests for Herpes viruses were also negative. These results seemed to us inconclusive, and since the radiological characteristics were highly suggestive for a malignant process, we decided to perform other biopsies in the deep lobe of the parotid gland. Hence, after having obtained the informed consent, the surgery was performed without any intra- and postoperative complications. The results were comparable to those of the first biopsies, with no evidence of a malignant process. At this point, we considered the possibility that the lesion could be an IPT; therefore, a rheumatologist was involved in the management of this patient. He required the evaluation of immunoglobulin G4 (IgG4) both in serum and in the bioptic samples, and examinations were negative. Nevertheless, the patient was treated with intravenous high-dose prednisone (500 mg) once a week for 6 weeks, which was tapered off over 2 weeks, plus intravenous cyclophosphamide 1,000 mg every 2 weeks for a total dose of 7 g. The treatment was well tolerated by the patient, with complete recovery of the trigeminal neuralgia and partial recovery of the facial palsy (grade II according to the House–Brackmann grading system). Moreover, the right effusive otitis media was solved. Three months after the conclusion of the treatment, a gadolinium-enhanced MRI showed a complete radiological response ( Fig. 2 ). Another MRI at 9 months follow-up showed no recurrence.

## Discussion

The IPT is a rare pathology of unknown etiology characterized histologically by acute and chronic inflammation, with varying proportions of lymphocytes, plasma cells, histiocytes, and fibrocytes. 3

Several theories have been proposed to explain the pathogenesis of IPTs, including trauma and surgical inflammation, autoimmune response (in particular IgG4 disease), and infections. 4

IPTs may affect any site of the body but most commonly the lung and the orbit. Another possible site of disease is the skull. In 70% of cases, the anterior skull base is affected, with presentation symptoms being pain (57%), vision change (36%), and hearing loss (33%). 5

Our patient presented with symptoms and radiological findings related to inflammation of the trigeminal and facial nerves with compression of the Eustachian tube that caused effusive otitis media. To the best of our knowledge, no previous cases reporting the involvement of these nerves have been described yet.

Imaging features in our case are those commonly described in the literature and consist of a mass with low intensity on both T1- and T2-weighted images, which reflect the fibrotic nature of these lesions, with homogeneous enhancement after contrast injection ( Fig. 1 ). Some authors have reported cases of multifocal IPTs; we performed a PET-CT scan to characterize the lesion, and there was no evidence of additional hypermetabolic foci.

Imaging features of IPTs may mimic malignant tumors, 4 and the final diagnosis is of exclusion by means of multiple biopsies, as in our case. As a matter of principle, the biopsy should be performed as a first procedure to avoid surgical overtreatment. Recently, some authors reported IPTs of the liver, pancreas, lung, and other organs, with characteristics similar to IgG4-related sclerosis disease as they involved lymphoplasmacytic infiltration and high IgG4+ plasma cell infiltrates. 6 Also, in the head and neck region, IgG4-related IPTs are reported. 7 Despite our efforts to demonstrate an involvement of IgG4 + , both the histopathological expression and blood level of our patient were not relevant.

As proposed by many authors, the first-line treatment of IPT is corticosteroid therapy, with different protocols that are reviewed by Ferri et al. 8 Comparing the first and second treatments that had been administered to the patient with the protocols listed in the table in Ferri et al's study, it is clear how the first therapy was not adequate to solve the pathology. We added the cyclophosphamide treatment as performed in other autoimmune diseases and other types of IPTs. 9

Low-dose radiation therapy is generally recommended in patients who are unresponsive or do not tolerate steroid treatment.

A complete or gross total resection should be limited to nonresponding symptomatic patients to solve or at least lessen the symptoms.

Clinical and radiological follow-up is of considerable importance due to the evidence of lymphomatous transformation in 10% of cases. 10

## Conclusion

IPT is a rare disease that must be reminded among the tumorlike lesions of the skull base since it is responsive to medical treatment. Imaging features are unspecific, and the bioptic diagnosis may avoid surgical overtreatment of the patient.
